# Health literacy and chronic disease prevalence: age-group differences in Zhejiang, China

**DOI:** 10.3389/fpubh.2025.1602658

**Published:** 2025-09-23

**Authors:** Qiuhua Zhang, Zishuo Huang, Tingke Xu, Chun Chen, Yue Xu, Dingming Yao, Xiujing Hu, Heni Chen, Yin Dong, Xuehai Zhang, Xiangyang Zhang

**Affiliations:** ^1^Department of Health Information, Sir Run Run Shaw Hospital, Zhejiang University School of Medicine, Hangzhou, Zhejiang, China; ^2^School of Public Health, Fudan University, Shanghai, China; ^3^NHC Key Laboratory of Health Technology Assessment, Fudan University, Shanghai, China; ^4^School of Medical Humanities and Management, Wenzhou Medical University, Wenzhou, Zhejiang, China; ^5^Zhejiang Center for Disease Control and Prevention (Zhejiang CDC), Hangzhou, Zhejiang, China; ^6^Health Community Group of Yuhuan People’s Hospital, Wenzhou, China; ^7^First Affiliated Hospital of Wenzhou Medical University, Wenzhou, Zhejiang, China

**Keywords:** middle-aged adults, older adults, health literacy, chronic diseases, cross-sectional studies

## Abstract

**Background:**

Health literacy (HL), a key factor in chronic disease prevention, enables individuals to better understand, manage, and respond to health risks. While HL’s influence on health outcomes is established, limited research has explored how its association with chronic disease prevalence varies across age groups, particularly between middle-aged adults and young-older adults.

**Methods:**

We used data from the 2023 China Health Literacy Survey (CHLS) in Zhejiang, which included 37,214 individuals aged 45 and older, collected through a stratified multistage probability sampling design. Participants were categorized into two age groups: middle-aged adults (45–59 years old) and young-older adults (60–69 years old). HL levels and the presence of chronic diseases were assessed. Chi-square test and multinomial logistic regression analyses were conducted to examine age-specific associations between HL and chronic disease status: (no chronic disease, one chronic disease, two or more chronic diseases), controlling for demographic covariates.

**Results:**

Among 37,214 participants, 20.3% had adequate HL, with a clear inverse relationship between HL and chronic disease status. Adequate HL was associated with significantly lower odds of having a single chronic disease among middle-aged adults (OR = 0.904, 95% CI: 0.836–0.978), though no such association was observed for two or more chronic diseases (multimorbidity). Furthermore, HL showed no significant association with chronic disease status across young-older populations. Consistent with these primary findings, results from all three HL dimensions—knowledge and attitudes, behavior and lifestyle, and health-related skills—aligned with this pattern.

**Conclusion:**

The study identified age-related differences in the link between HL and chronic disease. Among middle-aged adults, adequate HL was associated with significantly lower odds of a single chronic condition though not with multimorbidity, while no significant HL-chronic disease association existed in young-older populations. These findings underscore the need for targeted HL interventions tailored to middle-aged populations to mitigate early chronic disease onset.

## 1 Introduction

Chronic diseases—often long-lasting and with considerable impacts on global health—account for approximately 41 million deaths annually, representing 74% of all deaths worldwide (1). Older adults are particularly affected, experiencing not only higher mortality rates but also greater disability and diminished quality of life compared to younger populations (2–4). As the country with the world’s largest aging population, China faced substantial challenges in managing its chronic disease status. National statistics highlighted a significant age disparity: 69.8% of middle-aged adults have chronic conditions, whereas the prevalence rises sharply to 83.1% among older adults (5). This gap underscores the greater vulnerability of older adults in managing chronic diseases.

To address the significant age disparity in chronic disease prevalence, particularly the heightened vulnerability and potential management challenges faced by older adults, prevention strategies must start earlier in life and evolve beyond reliance on passive approaches. While existing measures like early screening and integrated care systems represent crucial steps towards early intervention (6), their effectiveness can be hampered among populations with limited health awareness. These approaches often require individuals to proactively seek services and adhere to recommendations—a significant barrier for those lacking knowledge or confidence, leading to low engagement and suboptimal outcomes. This limitation underscores the urgent need for a paradigm shift towards active strategies that proactively empower individuals through enhanced self-management skills and personal agency, thereby bridging the gap for vulnerable groups.

Promoting self-management among middle-aged and older adults is critically dependent on improving health literacy (HL) (7, 8). Defined as the capacity to obtain, understand, and use health information to make informed health decisions (9), HL encompasses three core domains essential for effective self-care: (1) knowledge and attitudes: grasping key health concepts and understanding preventive measures; (2) behavior and lifestyle: adopting and maintaining healthy practices, such as balanced nutrition, regular exercise, and proper hygiene; and (3) health-related skills: effectively navigating healthcare information and services. Strengthening these interconnected domains equips individuals with the foundational tools necessary for proactive health management.

Evidences have indicated distinct differences in HL and health-seeking behaviors between middle-aged and older adults. Middle-aged individuals generally exhibit higher HL levels; for instance, a Korean study documented significantly higher scores (12.43 vs. 8.34) compared to their older counterparts (10). Middle-aged individuals also tend to demonstrate greater initiative in health management, actively seeking information, participating in wellness activities, and utilizing healthcare services (11). Conversely, older adults frequently encounter significant barriers related to HL and self-management, such as difficulty understanding complex medical information or treatment plans and lower confidence in communicating with healthcare providers (12). These barriers potentially limit the effectiveness of conventional interventions (13).

Although previous studies have demonstrated a correlation between HL and chronic disease outcomes among adults aged 45 and above (14–16)—with higher HL being associated with healthier lifestyle choices, earlier detection of symptoms, and improved self-management, all of which may reduce overall disease burden—important age-related heterogeneities remain poorly understood. Not only do significant disparities exist in both chronic disease prevalence and HL levels between middle-aged and older adults, but it also remains unclear whether the association between HL and chronic disease status or management effectiveness varies across different age groups. This underscores the need to examine potential effect modifications by age within the HL–chronic disease relationship.

More importantly, given the trend of earlier chronic disease onset and the growing emphasis on primary and secondary prevention, there is a compelling rationale for focusing on younger older adults—specifically those aged 60 to 69 (17). Research within this subgroup carries heightened scientific and practical relevance, particularly for designing targeted early-intervention strategies. Nonetheless, this age segment remains substantially understudied. To address this gap and better capture age-specific variations, we stratified the sample into two groups: middle-aged adults (45–59) and young-older adults (60–69), thereby enabling a more nuanced understanding of how HL influences chronic disease outcomes during the critical transition into older age.

Employing a cross-sectional design, this study analyzed data from the 2023 China Health Literacy Survey (CHLS) (Zhejiang regional sample) to examine the association between HL and self-reported chronic disease condition among middle-aged adults and young-older adults in Zhejiang Province. Through age-stratified analyses, we independently assessed the HL-chronic disease relationship within each group. This approach delineates how HL accompanies disease burden across life stages, providing foundational evidence for targeted interventions and chronic disease prevention strategies in aging populations.

## 2 Methods

### 2.1 Study design

This study is a cross-sectional analysis utilizing data from the Zhejiang Province segment of the 2023 CHLS, a nationwide initiative aimed at assessing HL across Chinese populations. While CHLS targeted individuals aged 15–69 years who had resided in selected areas for at least 6 months, the current study focuses exclusively on adults aged 45 and older, aligning with the study’s objective to explore age-specific associations between HL and chronic disease prevalence (18, 19).

All participants received full information about the survey’s purpose, procedures, confidentiality protections, and contact details for the research team. Written informed consent was obtained prior to participation, in accordance with ethical standards approved by the Research Ethics Committee of the Zhejiang Provincial Center for Disease Control and Prevention.

Investigators completed standardized training sessions prior to data collection, covering questionnaire content, interviewing protocols, sampling methods, and quality assurance procedures. To ensure data integrity, quality checks—including random supervisory audits, onsite validations, and logic screening—were implemented at both local and provincial levels throughout the survey period.

### 2.2 Sample selection

The minimum sample size per layer carried out by each county (city, district) is calculated as *N* = $\frac{Z^{2}\times p\times (1-p)}{\delta^{2}}$× deff, where: N is the required sample size per stratum, Z is the standard normal deviate corresponding to the desired confidence level (1.96 for 95% confidence), p is the estimated proportion of individuals with adequate HL (0.3836, based on the 2022 estimate for Zhejiang Province),*δ* is the absolute error, calculated as 15% of p (i.e., δ = 0.15 × 0.3836 = 0.05754), deff is the design effect, set to 1 under the assumption of simple random sampling. Using these parameters, the minimum sample size per stratum was calculated to be N = 274. The sampling strategy employed a two-tier stratification based on urban and rural residency, ensuring representation across different geographic and socioeconomic contexts. To account for potential non-response and invalid questionnaires, a 10% adjustment was applied. Thus, the adjusted sample size per county (city or district) was: 274 participants × 2 strata ÷ (1–0.10) = 609. For operational convenience and to ensure divisibility during field implementation, the final sample size was rounded up to 640 participants per county (city or district).

The investigation encompassed all 90 administrative divisions (counties, cities, districts) within Zhejiang Province, commencing in September 2023 and concluding by November 2023. The sampling strategy adhered to national standards and consisted of four distinct phases: (1) employing a stratified multistage probability sampling method proportional to population size, selecting four townships per division; (2) using this method within townships to choose two communities (villages); (3) randomly sampling 100 households within each community (village); and (4) conducting in-person interviews using Kish’s grid to select one individual per household. Each community (village) aimed to survey a minimum of 80 participants to meet the study’s requirements. The study focused on middle-aged and older adults (aged 45 and above) in Zhejiang Province, yielding a final sample size of 37,214 individuals after excluding cases with missing data.

### 2.3 Measurements

The dependent variable, chronic disease status, was assessed using the question: “Presently, do you have any of the following chronic diseases?.” The listed conditions included hypertension, heart disease, cerebrovascular diseases, diabetes, malignant tumors, and others. Based on participant responses, chronic disease status was categorized into three groups: (1) no chronic disease, (2) single chronic disease, and (3) two or more chronic diseases (multimorbidity).

HL was appraised using the Chinese Health Literacy Scale, developed by the Chinese Center for Health Education. The maximum total score of the scale is 66 points, with the maximum total scores of the three dimensions being 28 (knowledge and attitudes), 22 (behavior and lifestyle), and 16 (health-related skills) points. Scores equal to or greater than 53 out of 66 (i.e., ≥80%) were classified as indicating adequate HL, while scores below 53 were considered limited. The same classification principle—using the 80% threshold—was applied individually to each of the three dimensions. The scale demonstrates robust reliability, with a Cronbach’s alpha coefficient of 0.95 and a Spearman-Brown coefficient of 0.94 (20).

The covariates were defined according to the previous studies (19, 21) and were as follows: gender (male, female), marital status (married, single), educational level (less than junior high school, junior/senior high school, college or above), occupation (public servant including civil servants, teachers, medical professionals, and other personnel affiliated with public institutions or non-public servant including students, farmers, manual laborers, private-sector employees, and others), Hukou (local, non-local), family size (1–2, 3–5, 6–), and annual household income (less than $100,000, $100,000 or more), and smoking (yes, no).

### 2.4 Statistical analysis

The data were analyzed using SPSS Statistics, version 26.0, with a significance level set at *p* < 0.05. Initially, a descriptive analysis was conducted to present the frequency and proportion of all variables (refer to Table 1). Chi-square test was then employed to assess the risk of chronic diseases across different categorical variables. Following this, multinomial logistic regression was utilized to investigate the relationship between HL and chronic condition among middle-aged adults and young-older adults adjusting by covariates including gender, marital status, educational attainment, occupation, Hukou, family size, annual household income, and smoking status, with a specific focus on age-related patterns. Lastly, we examined the association between the three dimensions of health literacy-Knowledge and attitudes, Behavior and lifestyle, and Health-related skills-and the presence of chronic conditions, with the purpose of ensuring the precise results of the study.

**Table 1:** The chronic condition by participant characteristics in Chinese middle-aged adults and young-older adults in 2023.

Variables	*N* (%)	Number of chronic diseases			χ^2^
		0	1	≥2	
Total	37,214 (100.0)	22,730 (61.1)	11,436 (30.7)	3,048 (8.2)	
Gender					
Male	17,876 (48.0)	10,523 (46.3)	5,820 (50.9)	1,533 (50.3)	71.181^***^
Female	19,338 (52.0)	12,207 (53.7)	5,616 (49.1)	1,515 (49.7)	
Age					
45–59	21,994 (59.1)	15,520 (68.3)	5,475 (47.9)	999 (32.8)	2262.373^***^
60–69	15,220 (40.9)	7,210 (31.7)	5,961 (52.1)	2,049 (67.2)	
Marital status					
Married	32,564 (87.5)	20,020 (88.1)	9,975 (87.2)	2,569 (84.3)	36.542^***^
Single	4,650 (12.5)	2,710 (11.9)	1,461 (12.8)	479 (15.7)	
Educational level					
Less than junior high school	14,633 (39.3)	8,045 (35.4)	5,010 (43.8)	1,578 (51.8)	481.750^***^
Junior/Senior high school	19,513 (52.4)	12,531 (55.1)	5,672 (49.6)	1,310 (43.0)	
College or above	2,154 (9.5)	2,154 (9.5)	754 (6.6)	160 (5.2)	
Occupation					
Public servant	2,667 (7.2)	1,747 (7.7)	735 (6.4)	185 (6.1)	24.127^***^
Non-public servant	34,547 (92.8)	20,983 (92.3)	10,701 (93.6)	2,863 (93.9)	
Family size					
1–2	15,624 (42.0)	8,828 (38.8)	5,227 (45.7)	1,569 (51.5)	419.184^***^
3–5	18,552 (49.9)	12,232 (53.8)	5,163 (45.1)	1,157 (38.0)	
6–	3,038 (8.2)	1,670 (7.3)	1,046 (9.1)	322 (10.6)	
Income (Dollar)					
0–9,9,999	20,488 (55.1)	12,002 (52.8)	6,549 (57.3)	1,937 (63.5)	158.101^***^
100,000–	16,726 (44.9)	10,728 (47.2)	4,887 (42.7)	1,111 (36.5)	
Hukou					
Local	35,916 (96.5)	21,811 (96.0)	11,119 (97.2)	2,986 (98.0)	57.365^***^
Non-local	1,298 (3.5)	919 (4.0)	317 (2.8)	62 (2.0)	
Smoking					
Yes	11,727 (31.5)	6,704 (29.5)	3,931 (34.4)	1,092 (35.8)	112.580^***^
No	25,487 (68.5)	16,026 (70.5)	7,505 (65.6)	1,956 (64.2)	
HL					
Adequate	7,559 (20.3)	5,136 (22.6)	1,996 (17.5)	427 (14.0)	205.765^***^
Limited	29,655 (79.7)	17,594 (77.4)	11,436 (82.5)	3,048 (86.0)	

*p < 0.05; **p < 0.01; ***p < 0.001.

## 3 Results

Table 1 presented the distribution of chronic condition status across key sociodemographic variables, with a particular focus on HL. Among the total sample of 37,214 individuals, 20.3% demonstrated adequate HL, while the remaining 79.7% had limited HL. The prevalence of chronic conditions increased markedly among those with limited HL. Specifically, among individuals with no chronic conditions (*n* = 22,730), 22.6% had adequate HL and 77.4% had limited HL. In contrast, among those with one chronic condition (*n* = 11,436), the proportion with adequate HL dropped to 17.5%, and among those with multimorbidity (*n* = 3,048), only 14.0% had adequate HL while a striking 86.0% had limited HL. This gradient suggests a clear inverse relationship between HL and chronic disease burden.

Table 2 presented results from multinomial logistic regression models stratified by age group. Among middle-aged adults, adequate HL was significantly associated with lower odds of having one chronic disease (OR = 0.904, 95% CI: 0.836–0.978, *p* < 0.05). However, this protective association was not statistically significant for having multimorbidity. In the young-older age group, the association between HL and chronic disease was weaker and not statistically significant. These results indicate that adequate HL may help reduce the risk of developing a single chronic condition, particularly in middle-aged adults.

**Table 2:** Association between HL and chronic condition in middle-aged adults and young-older adults.

Variables	OR (95% CI)	
	Middle-aged adults	Young-older adults
One chronic disease vs no chronic disease		
Gender (ref: Female)		
Male	1.227^***^ (1.127, 1.335)	1.043 (0.944, 1.152)
Marital status (ref: Single)		
Married	1.042 (0.939, 1.156)	1.037 (0.939, 1.146)
Educational level (ref: College or above)		
Less than junior high school	1.351^***^ (1.179, 1.550)	1.253 (0.993, 1.581)
Junior/Senior high school	1.087 (0.961, 1.229)	1.128 (0.901, 1.411)
Occupation (ref: Non-public servant)		
Public servant	0.968 (0.848, 1.106)	1.314^**^ (1.086, 1.590)
Family size (ref: 6–)		
1–2	0.895 (0.782, 1.023)	1.037 (0.921, 1.168)
3–5	0.780^***^ (0.687, 0.886)	0.940 (0.834, 1.061)
Income (ref: 100,000–)		
0–99,999	1.005 (0.941, 1.074)	0.951 (0.878, 1.030)
Hukou (ref: Non-local)		
Local	1.274^**^ (1.091, 1.488)	1.304^*^ (1.012, 1.681)
Smoking (ref: No)		
Yes	1.175^***^ (1.074, 1.285)	1.052 (0.949, 1.167)
HL (ref: Limited)		
Adequate	0.904^*^ (0.836, 0.978)	0.970 (0.868, 1.085)
Multimorbidity vs. no chronic disease		
Gender (ref: Female)		
Male	1.628^***^ (1.366, 1.940)	0.797^**^ (0.685, 0.927)
Marital status (ref: Single)		
Married	0.782^*^ (0.647, 0.947)	0.963 (0.838, 1.107)
Educational level (ref: College or above)		
Less than junior high school	1.840^***^ (1.360, 2.490)	1.155 (0.832, 1.603)
Junior/Senior high school	1.179 (0.892, 1.557)	0.908 (0.662, 1.246)
Occupation (ref: Non-public servant)		
Public servant	1.090 (0.819, 1.451)	1.480^**^ (1.132, 1.934)
Family size (ref: 6–)		
1–2	0.676^**^ (0.527, 0.866)	1.017 (0.859, 1.204)
3–5	0.526^***^ (0.417, 0.665)	0.881 (0.741, 1.047)
Income (ref: 100,000–)		
0–9,9,999	1.247^**^ (1.086, 1.433)	1.011 (0.900, 1.135)
Hukou (ref: Non-local)		
Local	1.620^*^ (1.123, 2.337)	1.432 (0.970, 2.116)
Smoking (ref: No)		
Yes	1.245^*^ (1.044, 1.485)	1.276^**^ (1.091, 1.494)
HL (ref: Limited)		
Adequate	0.935 (0.791, 1.104)	0.857 (0.724, 1.015)

*p < 0.05; **p < 0.01; ***p < 0.001.

Table 3 further explored the relationship between specific dimensions of HL and chronic disease. Among middle-aged adults, all three HL dimensions—knowledge and attitudes, behavior and lifestyle, and health-related skills—were significantly associated with reduced odds of having one chronic disease (ORs ranging from 0.895 to 0.906, *p* < 0.01). However, none of the HL dimensions showed significant associations with having multimorbidity in either age group. These findings suggest that targeted improvements in HL components may be particularly effective in preventing the onset of chronic disease, though their impact may diminish as disease burden increases.

**Table 3:** Association between HL dimensions associated and chronic diseases in middle-aged adults and young-older adults.

HL Dimensions	OR (95% CI)	
	Middle-aged adults	Young-older adults
One chronic disease vs. no chronic disease		
Knowledge and attitudes (ref: Limited)	0.897^**^ (0.837, 0.962)	0.986 (0.901, 1.079)
Behavior and lifestyle (ref: Limited)	0.906^**^ (0.840, 0.976)	1.026 (0.931, 1.130)
Health-related skills (ref: Limited)	0.895^**^ (0.828, 0.968)	1.032 (0.925, 1.152)
Multimorbidity vs. no chronic disease		
Knowledge and attitudes (ref: Limited)	0.978 (0.846, 1.132)	0.900 (0.788, 1.029)
Behavior and lifestyle (ref: Limited)	1.059 (0.907, 1.236)	0.973 (0.844, 1.122)
Health-related skills (ref: Limited)	0.954 (0.809, 1.125)	0.936 (0.794, 1.103)

*p < 0.05; **p < 0.01; ***p < 0.001.

## 4 Discussion

To our knowledge, this is the first study utilizing CHLS data to examine age-stratified associations between HL and self-reported chronic disease status among middle-aged and older adults. Cross-sectional analyses revealed nuanced patterns: stratified by age, the inverse HL-chronic disease association remained significant only for middle-aged adults with a single chronic condition, whereas no significant relationships emerged between HL and chronic disease status (single or multiple) in young-older adults.

Research conducted out of China indicated that HL strongly correlated with chronic disease prevalence, especially among older adults—a pattern consistently observed in cross-sectional studies from the United States, Switzerland, and Korea, where limited HL predicts higher rates of conditions like arthritis and hypertension (22–24). Paradoxically, despite global evidence supporting the protective role of HL in chronic disease prevention, our age-stratified analysis revealed a more complex picture. Among middle-aged adults, higher HL was significantly associated with reduced prevalence of single chronic conditions, but showed no significant relationship with multiple conditions. In contrast, among older adults, HL was not significantly associated with either single or multiple chronic conditions. This discrepancy raises a critical question: Why does HL appear effective in mitigating early-stage disease burden among middle-aged individuals, yet fail to predict chronic disease outcomes in China’s aging population?

This discrepancy may be partly explained by comparatively lower HL levels among older individuals. At present, there is no universally accepted framework for measuring HL in aging populations. Existing tools vary widely in structure and cultural relevance, limiting cross-study comparability. Generally, HL levels are higher in developed nations; for instance, studies show that 30.9% of older adults in Spain and 36.4% in Denmark have inadequate or problematic HL (25, 26). In contrast, a meta-analysis in China found that only 12.28% (95% CI: 10.70–13.95%) of the older adults possessed adequate HL, revealing a significant disparity (27). Although the Chinese Health Literacy Scale was a widely validated tool for assessing HL in adult populations, its applicability to older adults may be limited due to age-related cognitive variation and the digital divide. These factors could affect comprehension and response accuracy, potentially leading to misclassification, especially around the cut-off point for adequate HL.

Causes of low HL among Chinese older adults can be analyzed from both supply and demand perspectives. On the supply side, healthcare access remains limited, and dissemination of health-related information is insufficient. Many older adults experience difficulty obtaining timely and comprehensible medical guidance (28). On the demand side, personal capabilities and behaviors significantly influence HL levels. Educational attainment is generally low among older adults, which restricts their ability to understand scientific health information (29). Cognitive decline, commonly associated with aging, further impairs their capacity to process and apply health knowledge (27). Moreover, the digital divide—caused by unfamiliarity with internet tools and information technologies—limits access to online health resources and services, thereby hindering engagement with up-to-date health content (30). These interwoven challenges compound the problem of inadequate HL in this demographic.

Moreover, our findings revealed that HL was not significantly associated with multimorbidity in either middle-aged or older adults. This suggests that while HL may play a role in preventing single chronic conditions—particularly among middle-aged individuals—its effectiveness in addressing more complex disease conditions, such as multimorbidity, appears limited. While improved HL effectively prevents single chronic conditions in middle-aged adults, it demonstrates limited efficacy against multimorbidity due to its complex etiology. Addressing multimorbidity requires moving beyond self-management to establish integrated healthcare pathways.

In addition to HL, a range of covariates significantly influenced chronic disease outcomes. Among middle-aged adults, male gender and smoking were associated with higher odds of developing a single chronic condition, consistent with established behavioral risk patterns (31). Lower educational attainment was consistently linked to an increased risk of both single and multiple chronic conditions, underscoring the critical role of education in shaping health behaviors and healthcare access (32). Interestingly, smaller household size was linked to reduced odds of multimorbidity, possibly due to lower caregiving burdens or more autonomous health management (33). These findings underscore the importance of integrating sociodemographic and behavioral factors into chronic disease prevention strategies.

However, this study’s exclusive focus on young-old adults (60–69 years) constitutes a key limitation, as findings cannot be generalized to older-old adults (≥70 years). Consequently, the identified HL patterns and their association with chronic conditions are strictly representative of China’s youngest older adults cohort—a demographic with relatively higher digital adoption rates and educational attainment. While future research must investigate HL dynamics in older-old populations (as detailed in Limitations), the current findings offer actionable insights for this critical subgroup.

Given the age-specific associations between HL and chronic disease status, public health strategies should adopt a life-course approach to HL enhancement. For middle-aged adults, interventions should emphasize proactive chronic disease prevention and the development of self-management skills. HL programs must focus on recognizing early symptoms, interpreting health information, and making informed lifestyle choices. These initiatives are best delivered through structured environments such as workplace health programs, community clinics, and digital platforms—ensuring broad accessibility and sustained engagement to delay or reduce the incidence of initial chronic conditions. For younger-older adults (e.g., 60–69), strategies should extend beyond HL education to include structural and supportive measures. While improving HL remains useful, policies must also simplify clinical care pathways, integrate caregiver education, promote cognitive-friendly health communication, and ensure easy access to reliable health services. Special attention should be given to the use of visual aids, repeated health messaging, and integrated support systems that accommodate age-related cognitive changes. Across both groups, it is essential to address upstream determinants of HL—such as educational attainment, digital inclusion, and regional health infrastructure—to reduce disparities and ensure equitable impact. Integrating HL initiatives within multidisciplinary chronic disease management systems, including community-based team care and patient navigation programs, will help embed HL into broader prevention and long-term health sustainability efforts.

## 5 Conclusion

The study revealed an age-related disparity in the association between HL and chronic disease burden. Among middle-aged adults, adequate HL was significantly associated with lower odds of having one chronic condition, but showed no significant link with multimorbidity. In contrast, among older adults, HL was not significantly associated with either single or multiple chronic conditions. These findings suggested that while promoting HL may be effective for preventing the initial onset of chronic disease in middle-aged populations, broader structural and policy-level interventions may be necessary to address disease burden in young-older adults.

## 6 Limitation

Despite the valuable insights provided by this study, several limitations should be acknowledged. First, as a cross-sectional investigation, the findings illustrate associations rather than causation, which precludes definitive conclusions regarding the directional relationship between HL and the prevalence of chronic diseases among middle-aged and older adults. Future research should adopt longitudinal or cohort designs to more accurately explore potential causal pathways. Second, the age range of older adult participants was restricted to 60–69 years, which may not fully capture the HL dynamics across the broader older adults. Subsequent studies should consider including individuals over 70 years of age to enhance generalizability. Lastly, the sample was drawn exclusively from Zhejiang Province, potentially limiting the external validity of the results. To better understand regional disparities and ensure a nationally representative perspective, future studies should incorporate diverse geographic areas across China.

## Data Availability

The original contributions presented in the study are included in the article/supplementary material, further inquiries can be directed to the corresponding author/s.
